# The effect of exercise prescription of primary care physician on the quality of life in patients

**DOI:** 10.1080/17571472.2018.1464731

**Published:** 2018-04-25

**Authors:** Hakan Yaman, Emrah Atay

**Affiliations:** a Independent Scholar; b Faculty of Sports Sciences, Mehmet Akif Ersoy University, Burdur, Turkey

**Keywords:** Family practice, primary health care, exercise prescription, prevention, health promotion, quality of life, Turkey

## Abstract

**Purpose:**

The aim of this study was to examine the effect of exercise prescribed by primary care physicians (PCPs) on the quality of life (QoL) of elderly people.

**Method:**

Randomisation was performed at PCPs level from 16 primary healthcare centers. Patients were divided into intervention and control groups. Both groups of physicians received theoretical training (14 h), and the intervention group received additional practical training on exercise prescription (10 h). Patients in the intervention group were prescribed endurance, flexibility, balance, and strength exercises and were given training packs. QoL was measured using Short Form-36. Measurements were taken at the beginning of the study, after the 3rd month, and at the end of the 6th month to evaluate the effectiveness of the intervention.

**Results:**

The age of participants (Intervention group *n* = 69, Control group *n* = 110) was 57.68 ± 5.08 years. At the end of the study, physical function, physical role function, social role function, mental health, vitality, general health perception, and emotional role function scores increased and body pain scores decreased in the intervention group. Significant differences (*p* < 0.05) between the intervention and control groups were observed for physical function, physical role function, body pain, mental health, vitality, and emotional role function scores but not for social role function or general health perception scores.

**Conclusions:**

Exercise prescriptions given by PCPs containing endurance, strength, flexibility, and balance exercises improve QoL in elderly people.

## Why this matters to me

This subject is important to me because physical activity is one of the most important pillars of health and well-being. Prescribing this important and free modality in an easy way to patients in daily practice is important in my opinion.

## Key message

Prescriptions for moderate intensity exercise, which included endurance, strength, flexibility and balance, components, increased most aspects of QOL of participants.

## Introduction

The aging process in humans impairs functional capacity and performance. Physical activity seems to be an appropriate instrument to overcome these obstacles by minimizing the changes in organ systems and enhancing the capabilities of the aging person [1]. Strong evidence suggests that physically active people suffer less from coronary heart disease, dyslipidemia, hypertension, stroke, type II diabetes, osteoporosis, obesity, and mental health In addition, enhancements in functional capacity, cognitive capability, and a decrease in the risk of falling have been observed. Sedentary lifestyle could increase the risk of colon, breast, prostate and lung cancer [2–5]. To attain these improvements the World Health Organization recommends a minimum of 150 min of moderate or 75 min of high intensity exercise per week is recommended for elderly people [3].

Physicians and health professionals play an important role during the promotion of exercise- and health-related activities. They possess a unique opportunity to counsel their patients and promote healthy behavior. A study by Atay et al. has shown that exercise prescription has increased physical activity and functional properties of adults and elderly people [6].

Quality of life (QoL) includes dimensions of physical, psychological, environmental, economical, and social well-being and personal beliefs of patients [7,8]; and is an outcome of multifactorial interactions in a patient’s life [9]. Improvement of functional properties such as sitting, standing, climbing steps, stretching, etc., which are related to cardiovascular, neuromuscular, and musculoskeletal systems, would have a positive impact on the QoL of the elderly person [10].

To our knowledge studies on the effect of exercise prescription in primary care setting on QoL setting are scarce [11]. The aim of this study was to examine the effect of exercise prescribed by primary care physicians (PCPs) on the QOL of people.

## Materials and method

This study originated from a PhD thesis, which has been published previously [6]. Details on inclusion/exclusion criteria, recruitment of participants, study design, and socio-demographic properties of participants have been described in this study [6]. Ethical approval was obtained from the Ethics Committee of Akdeniz University Faculty of Medicine (11.05.2006 and Nr: B.30.2.AKD.0.01.00.00/Etik-221) before starting this trial. Participants have been divided into intervention (IG) and control (CG) groups. The effect of the intervention was measured at 3 and 6 months.

Participants were sedentary and capable of physical activity. The cognitive score was at least 24 points (MMSE) [12] for every participant. All were cognitively in good condition. Participants were able to understand the recommendations and advice of the PCP. Health status was evaluated by The Physical Activity Readiness Questionary (PAR-Q) [13]. Sedentarity was assessed with The Brief Physical Activity Assessment Survey [14].

**Figure 1.**
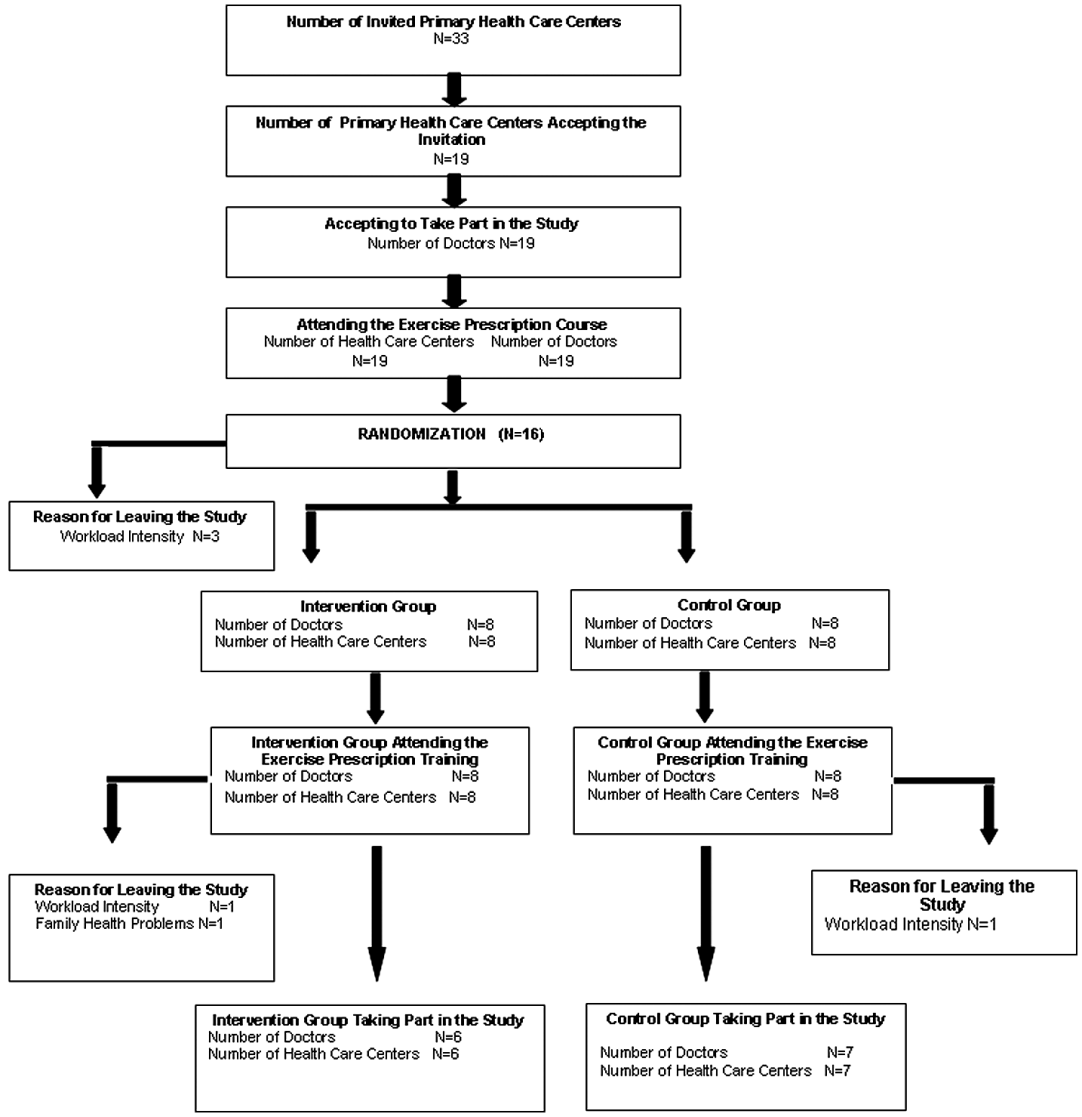
Consort diagram.

**Figure 2.**
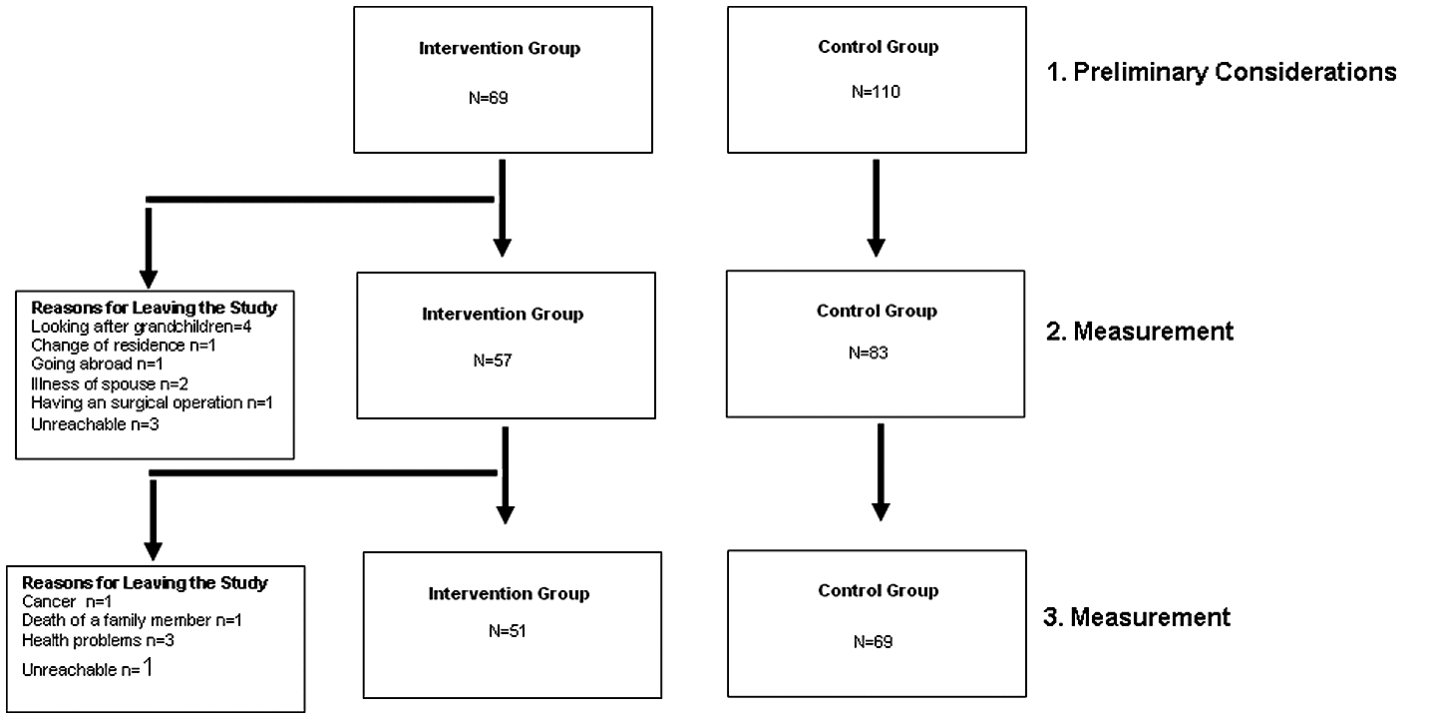
Consort diagram (cont.).

Nine-teen of 33 primary health care centers (PHCCs), agreed to participate in this study. After the first meeting, three centers left the study because of heavy workload. Sixteen PCPs from 16 PHCCs were randomized into the IG (*n* = 8) or CG (*n* = 8) [6]. Contamination was therefore avoided (Figures 1 and 2). The outcomes were assessed blindly.

PCPs of both groups received theoretical training (14 h), and PCPs of the IG were given further practical advice (10 h) regarding exercise prescription. Subjects from the course are on motivational interviewing, behavioral change, approaches on physical activity, exercise and methods, and questionnaires and scales. The practical part included application of Rate of Perceived Emotion Scale, speaking test, elastic band, flexibility exercises, balance exercises, and exercise prescription.

*Methods on survey application: Both groups PCPs received a ‘Physicians Handbook’ after the course. PCPs of the IG received additional materials for exercise prescription and training packs for the patients, which included a brochure on exercise, Rate of Perceived Exertion (RPE-Borg Scale), and Thera-Band elastic bands, etc.

PCPs in the IG prescribed exercise with an appropriate balance between endurance, flexibility, balance, and strength exercises. Endurance was prescribed 3–5 days a week and strength, balance, and flexibility were prescribed 2–3 days a week. Elastic bands enabled patients in the IG to perform strength exercises. Each daily exercise session was recommended to start with a warm-up session and concluded with a cool-down session. Exercise duration and volume was gradually increased to create a training effect. To estimate the intensity of the exercise, the RPE scale was used [15]. The exercise intensity was set at a medium level (RPE scale 12–14 points). The exercises were monthly prescribed and the exercise prescriptions were renewed every month. The increase in exercise time of exercise prescription were arranged according to participants’ verbal answers*.*

General characteristics of the exercise prescriptions were as follows: Endurance 3–5 times per week and 15–30 min of duration; strength 2–3 times per week and in 13 sets; flexibility 2–3 times per week, in 13 sets and in 10–30 s each exercise; balance 2–3 times per week, 13 sets, and in 15–30 s each exercise.

Patients in the IG received thorough counseling on exercise, whereas those in the CG only received 5–10 min of instruction. In addition, patients in the CG did not receive an exercise prescription or exercise pack.

QOL has been measured using the Turkish version of health outcome measure Short Form (SF)-36 [16,17]. This measure consists of 36 items and evaluates QOL-related issues of the last 4 weeks. The SF-36 covers the following eight domains: physical function, social function, physical role, emotional role, mental health, vitality, body pain, and general health perception. The range of scores is 0–100 and higher scores reflect better QOL.

Measurements have been performed at baseline, 3rd and 6th month.

## Statistical analysis

The Shapiro–Wilk and Levene tests were used to evaluate the distribution and variance homogeneity of data. Multiple 3 × 2 (time × group) repeated-measures analyses of variance (ANOVAs) were performed to determine differences within and between groups over time. When statistical differences were observed between the groups at study entry, an analysis of covariance (ANCOVA) was performed on outcome variables at the conclusion of the study. The covariate used was the baseline value for each participant for the particular outcome variable being analyzed. These analyses are exploratory and there is no single main outcome. Statistical results have been divided by design effect coefficient (1 + (*m* – 1)*ρ*) = 1.65. Where m was the number of observation in each cluster (*n* = 14) and *ρ* was the ICC retrieved from literature for the subdomain ‘vitality’ as 0.05 [18]. The level of significance was set to *p* = 0.05.

## Results

The age of participants (*n* = 179) was 57.68 ± 5.08 years. At the beginning, 69 were assigned to IG and 110 to CG. The study concluded with 120 participants [IG (*n* = 51) and CG (*n* = 69)]. Reasons for leaving the study, socio-demographic characteristics, study design and study limitations have been described in another article [6].

At the beginning of this study were significant differences (*p* < 0.05) general health Perception, mental health scores which are subscales of QOL. The QOL scores of both groups at baseline are shown in Table 1.

**Table 1.** Short Form-36 scores at baseline.

SF-36 Dimensions (score)	IG *n* = 69 (mean ± SD)	CG *n* = 110 (mean ± SD)	*t*(*p*) [*Z*(*p*)]
Physical Function	81.67 ± 11.93	84.91 ± 12.13	**−1.07 (>0.05)
Physical Role	86.23 ± 32.24	80.91 ± 33.34	**0.64 (>0.05)
Body Pain	26.09 ± 22.31	31.36 ± 22.93	**−0.92 (>0.05)
General Health Perception	54.86 ± 9.15	50.41 ± 11.83	*1.71 (>0.05)
Vitality	53.99 ± 11.99	52.09 ± 12.33	**0.62 (>0.05)
Social Role Function	47.10 ± 11.37	43.52 ± 12.64	**1.19 (>0.05)
Emotional Role Function	74.88 ± 41.38	63.94 ± 42.87	**1.03 (>0.05)
Mental Health	57.04 ± 8.07	52.95 ± 10.38	*1.79 (>0.05)

Notes: IG, Intervention Group; CG, Control Group; RM-ANOVA, repeated-measures analysis of variance; RM-ANCOVA, repeated-measures analysis of covariance.

**Two-way RM-ANOVA Test *Two-way RM-ANCOVA Test.

Several domains of the SF-36 changed because of the exercise intervention. Physical function, physical role function, body pain, mental health, vitality, and emotional role function scores were significant differences (*p* < 0.05) but not for social role function or general health perception scores. Changes according to time, between groups, and group × time interactions are displayed in Table 2.

**Table 2.** Short Form-36, inter-group, time-dependent, and group-time changes in response to prescribed exercise.

	Baseline		3rd Month		6rd Month (Post-Intervention)		Times	Times-Group	Group
Subdivision points	IG (mean ± SD)	CG (mean ± SD)	IG (meanc ± SD)	CG (mean ± SD)	IG (mean ± SD)	CG (mean ± SD)			
Physical Function	81.67 ± 11.93	84.91 ± 12.13	86.52 ± 11.36	78.31 ± 13.57	94.80 ± 4.69	72.83 ± 10.34	*F*_*2.117*_ = *2.17, p* < *0.05*	*F*_*2.117*_ = *47.81, p* < *0.05*	**F_*1.118*_ = *18.40, p* < *0.05*
Physical Role Function	86.23 ± 32.23	80.91 ± 33.34	93.86 ± 19.64	83.13 ± 32.22	99.51 ± 3.50	93.16 ± 21.81	*F*_*2.117*_ = *16.12, p* < *0.05*	*F*_*2.117*_ = *0.14, p* > *0.05*	**F_*1.118*_ = *5.47, p* < *0.05*
Body Pain	26.09 ± 22.30	31.36 ± 22.93	23.86 ± 16.77	33.86 ± 19.81	13.53 ± 11.63	24.20 ± 13.76	*F*_*2.117*_ = *11.95, p* < *0.05*	*F*_*2.117*_ = *0.02, p* > *0.05*	**F_*1.118*_ = *11.18, p* < *0.05*
Social Role Function	47.10 ± 11.37	43.52 ± 12.64	44.30 ± 10.32	45.03 ± 11.20	53.43 ± 8.69	47.83 ± 12.49	*F*_*2.117*_ = *7.24, p* < *0.05*	*F*_*2.117*_ = *2.60,p* < *0.05*	**F_*1.118*_ = *1.95, p* = *0.05*
Mental Health	57.04 ± 8.07	52.94 ± 10.38	58.38 ± 9.47	57.78 ± 8.53	63.53 ± 4.50	57.91 ± 7.87	*F*_*2.116*_ = *84.20, p* < *0.05*	*F*_*2.116*_ = *5.16, p* < *0.05*	*F_*1.117*_ = *2.54, p* < *0.05*
Emotional Role Function	74.88 ± 41.38	63.93 ± 42.87	87.13 ± 28.70	77.51 ± 35.35	94.12 ± 12.83	82.61 ± 23.99	*F*_*2.117*_ = *6.90, p* < *0.05*	*F*_*2.117*_ = *0.14, p* > *0.05*	**F_*1.118*_ = *2.59, p* < *0.05*
Vitality	53.99 ± 1.99	52.09 ± 12.33	52.81 ± 11.15	45.84 ± 10.06	57.45 ± 6.96	55.22 ± 9.60	*F*_*2.117*_ = *11.59, p* < *0.05*	*F*_*2.117*_ = *2.92, p* < *0.05*	**F_*1.118*_ = *6.13, p* < *0.05*
General Health Perception	54.86 ± 9.15	50.41 ± 11.83	55.00 ± 7.07	52.89 ± 9.97	55.69 ± 5.92	55.07 ± 7.97	*F*_*2.116*_ = *67.20, p* < *0.05*	*F*_*2.116*_ = *0,13, p* > *0.05*	*F_*1.117*_ = *0.07, p* > *0.05*

Notes: IG, Intervention Group; CG, Control Group; RM-ANOVA, repeated-measures analysis of variance; RM-ANCOVA, repeated-measures analysis of covariance.

**Two-way RM-ANOVA Test *Two-way RM-ANCOVA Test,.

## Discussion

In this study, an exercise intervention that was prescribed by PCPs in real life PHCCs exhibited positive effects compared to the control group on physical function, physical role function, mental health, vitality, and emotional role function scores, which are subscales of QOL. Body pain increased in the IG.

The improvement in most domains of the QoL measure could indicate suitability of exercise prescription in PHCC’s to enhance physical activity and a healthy lifestyle [11]. These findings are similar to the study Kallings LV, et al. [11], which showed improvement in most dimensions of health-related QoL after six month exercise intervention. Another study in postmenoposal participants showed increase in QoL during a six months intervention [19]. In our study social role function and general health were not affected by exercise. Body pain increased in the IG.

Mobility and functionality seems to be important contributors to the QOL of aging people. An active life incorporating regular physical activity enhances performance and functional capacity [6]. Physiological and functional losses could be prevented with regular exercises. Functional properties have been shown to be related to QOL [10,20].

Regular physical activity prevents disease, promotes health, and improves QOL. Although this is well-known, participation in sport activities remain low [21].

This study shows that exercise prescription at the primary care level has benefits on QOL in patients. Sorenson et al. has reported similar results [22]. Several studies have reported benefits of exercise on QOL [23–25]; however, studies with exercise prescription as an intervention and their effect on QOL are rare [11,22].

Although exercise prescriptions are known to promote health [11], further studies are needed to elucidate the relationships with dose, gender, duration, morbidity, etc. [23,24,26].

The exercise prescriptions for elderly people should include an aerobic exercise component for optimal benefit [27]. Moderate intensity exercises are recommended to provide a safe and comfortable environment [23]. Functional abilities like walking, bending, balance, and stretching need to be particularly addressed [28].

Limitation of this study were the dropout rate of participants, which could have consequences on missing data of this study. But because of stringent inclusion criteria the dropout did not involve more older, frail and participants with lower Qol scores. Kallings LV, et al. reported similar dropout rate (38%) [11]. Correction for multiple testing have not been performed and the analyses of this study are therefore exploratory in nature. Because of the multi-dimensional nature of SF-36 measure, no single main outcome result could be achieved. The direct effect of the PCPs on the patients have not been analysed with nesting and multiple level modeling.

In conclusion, prescriptions for moderate intensity exercise, which were prescribed by PCPs and included endurance, strength, flexibility and balance, components, increased most aspects of QOL of participants.

## Disclosure statement

No potential conflict of interest was reported by the authors.

## Funding

This study was supported by the Akdeniz University Research Management Unit [Project number 2006.03.0122.006] and Thera-Band, GmbH, Hadamar Company, Germany.

## References

[1] Chodzko-Zajko WJ, Proctor DN, Fiatarone Singh MA, et al American college of sports medicine position stand. Exercise and physical activity for older adults. Med Sci Sports Exerc. 2009;41(7):1510–1530. PMID: 19516148 10.1249/MSS.0b013e3181a0c95c 19516148

[2] Atay E, Hekim M The effect of physical activity on health in adult individuals. Int Ref Acad J Sports. 2013;3(7):113–122.

[3] WHO Physical activity and older adults. [Internet]. [cited 2015 Feb 4]. Available from: http://www.who.int/dietphysicalactivity/factsheet_olderadults/en/

[4] Colditz GA, Cannuscio CC, Frazier AL Physical activity and reduced risk of colon cancer: implications for prevention. Cancer Causes Control. 1997;8:649–667. 10.1023/A:1018458700185 9242482

[5] Seth A Exercise prescription: what does it mean for primary care? Br J Gen Pract. 2014 Jan;64(618):12–13. 10.3399/bjgp14X676294 24567552 PMC3876165

[6] Atay E, Toraman NF, Yaman H Exercise prescription by primary care doctors: effect on physical activity level and functional abilities in elderly. Turk J Geriatr. 2014;17(1):77–85.

[7] Eriksson M, Hagberg L, Lindholm L, et al Quality of life and cost-effectiveness of a 3-yeartrial of lifestyle intervention in primary health care. Arch Intern Med. 2010;170(16):1470–1479. 20837834 10.1001/archinternmed.2010.301

[8] Bonomi AE, Patrick DL, Bushnell DM, et al Validation of the United States’ version of the World Health Organization Quality of Life (WHOQOL) instrument. J Clin Epidemiol. 2000;53:1–12. 10.1016/S0895-4356(99)00123-7 10693897

[9] Erci B, Demirdöğen O Predictors of quality of life in Turkish adult primary care patients. Holist Nurs Pract. 2013;27(4):233–238. 10.1097/HNP.0b013e318294e624 23774723

[10] Rejeski WJ, Mihalko SL Physical activity and quality life in older adults. J Gerontol A Biol Sci Med Sci. 2001;56(Supplement 2):23–35. PMID: 11730235 10.1093/gerona/56.suppl_2.23 11730235

[11] Kallings LV, Leijon M, Hellenius ML, et al Physical activity on prescription in primary health care: a follow-up of physical activity level and quality of life. Scand J Med Sci Sports. 2008;18(2):154–161. PMID. 17555539 10.1111/j.1600-0838.2007.00678.x

[12] Güngen C, Ertan T, Eker E, et al Reliability and validity of the standardized mini mental state examination in the diagnosis of mild dementia in Turkish population. Turk J Psychiat. 2002;13(4):273–281. PMID: 12794644. 12794644

[13] Thomas S, Reading J, Shephard RJ Revision of the physical activity readiness questionnaire (Par-Q). Can J Sport Sci. 1992;17:338–345. 1330274

[14] Marshall AL, Smith BJ, Bauman AE, et al Reliability and validity of a brief physical activity assessment for use by family doctors. Br J Sports Med. 2005;39:294–297. 10.1136/bjsm.2004.013771 15849294 PMC1725203

[15] Robertson RJ Perceived exertion for practitioners rating effort with the OMNI picture system. 1st ed Champaign: Human Kinetics; 2004 p. 53–62.

[16] Ware JE, Sherbourne CD The most 36 item short-form health survey (SF-36): I. conceptual framework and item selection. Med Care. 1992;30:473–483. PMID: 1593914 10.1097/00005650-199206000-00002 1593914

[17] Koçyiğit H, Aydemir Ö, Ölmez N, et al Short form-36 (SF-36): reliability and validity of the Turkish version. J Drugs Treatments. 1999;12:102–106.

[18] Elley CR, Kerse N, Arroll B, et al Effectiveness of counselling patients on physical activity in general practice: cluster randomised controlled trial. BMJ. 2003 Apr 12;326(7393):793 10.1136/bmj.326.7393.793 12689976 PMC153098

[19] Martin CK, Church TS, Thompson AM, et al Exercise dose and quality of life: a randomized controlled trial. Arch Intern Med. 2009;169(3):269–278. 10.1001/archinternmed.2008.545 19204218 PMC2745102

[20] Mavric F, Kahrovic I, Muric B, et al The effects of regular physical exercise on human body. Phys Cult. 2014;1:29–38.

[21] Earin E, Glasgow RE, Riley K Review of primary care-based physical activity intervention studies. J Fam Pract. 2000;19(2):158–168. PMID: 10718694. 10718694

[22] Sorensen J, Sorensen JB, Skovgaard T, et al Exercise on prescription: changes in physical activity and health-related quality of life in five Danish programmes. Eur J Public Health. 2010;21(1):56–62. PMID: 20371500. 20371500 10.1093/eurpub/ckq003

[23] Imayama I, Alfano CM, Bertram LAC, et al Effects of 12-month exercise on health-related of life: a randomized controlled trial. Prev Med. 2011;52:344–351. PMID: 21371498. 10.1016/j.ypmed.2011.02.016 21371498 PMC3086667

[24] DeVries NM, Van Ravensberg CD, Hobbelen JSM, et al Effects of physical exercise therapy on mobility, physical functioning, physical activity and quality of life in community-dwelling older adults with impaired mobility, physical disability and/or multi-morbidity: A meta-analysis. Ageing Res Rev. 2012;11:136–149. PMID: 22101330 10.1016/j.arr.2011.11.002 22101330

[25] Vuillemin A, Boini S, Bertrais S, et al Leisure time physical activity and health-related quality of life. Prev Med. 2005;41:562–569. PMID: 15917053 10.1016/j.ypmed.2005.01.006 15917053

[26] Aovagi Y, Park H, Park S, et al Habitual physical activity and health-related quality of life in order adults: interactions between the amount and intensity of activity. Qual Life Res. 2010;19:333–338. PMID: 20084463. 20084463 10.1007/s11136-010-9588-6

[27] McDermott AY, Mernitz H Exercise and older patients: prescription guidelines. Am Fam Physican. 2006;74(3):438–444. PMID: 16913163. 16913163

[28] Seco J, Abecia LC, Echevarri E, et al A long-term physical activity training program increase strength and flexibility, and improves balance in older adults. Rehabil Nurs. 2012;38:37–47. PMID: 23365004. 10.1002/rnj.64 23365004

